# Daylight Saving Time and Artificial Time Zones – A Battle Between Biological and Social Times

**DOI:** 10.3389/fphys.2019.00944

**Published:** 2019-08-07

**Authors:** Till Roenneberg, Eva C. Winnebeck, Elizabeth B. Klerman

**Affiliations:** ^1^Institute of Medical Psychology, Ludwig Maximilian University of Munich, Munich, Germany; ^2^Division of Sleep and Circadian Disorders, Brigham and Women’s Hospital, Harvard Medical School, Boston, MA, United States

**Keywords:** circadian, social jetlag, circadian misalignment, time zones, entrainment (light)

## Abstract

Many regions and countries are reconsidering their use of Daylight Saving Time (DST) but their approaches differ. Some, like Japan, that have not used DST over the past decades are thinking about introducing this twice-a-year change in clock time, while others want to abolish the switch between DST and Standard Time, but don’t agree which to use: California has proposed keeping *perennial* DST (i.e., all year round), and the EU debates between *perennial* Standard Time and *perennial* DST. Related to the discussion about DST is the discussion to which time zone a country, state or region should belong: the state of Massachusetts in the United States is considering switching to Atlantic Standard Time, i.e., moving the timing of its *social clock* (local time) 1 h further east (which is equivalent to perennial DST), and Spain is considering leaving the Central European Time to join Greenwich Mean Time (GMT), i.e., moving its social timing 1 h further west. A wave of DST discussions seems to periodically sweep across the world. Although DST has always been a political issue, we need to discuss the biology associated with these decisions because the circadian clock plays a crucial role in how the outcome of these discussions potentially impacts our health and performance. Here, we give the necessary background to understand how the *circadian clock*, *the social clock*, *the sun clock*, time zones, and DST interact. We address numerous fallacies that are propagated by lay people, politicians, and scientists, and we make suggestions of how problems associated with DST and time-zones can be solved based on circadian biology.

## Introduction

The issue of Daylight Saving Time (DST) is an indirect consequence of dividing the surface of Earth into time zones. After decades of measurements, this action was taken at a conference in Washington DC in 1884 to facilitate communication and travel between places with different sun times [as exquisitely recounted in Kehlmann (2009)]. Our Earth takes (at present) 24 h for one rotation. Notably, Earth’s day was not always 24 h. When the first biological clocks developed to organize physiology on a daily level (i.e., circadian clocks), probably something like 3 billion years ago in ancestors of today’s cyanobacteria (Dvornyk et al., 2003), days on Earth were 22 h or even shorter (Williams, 2000); days have lengthened by approximately 2 ms every century since. The current 24-h-day translates to an angular velocity of 4 min per longitudinal degree, so the Earth rotates by 15° every hour.

Since 1884, the world’s reference clock ticks on Observatory Hill in Greenwich east of London – it defines the zero meridian. Meridians are imaginary lines that run between the North and the South Pole and cut the earth into 360 even “apple slices.” Theoretically, time zones (for the social local clock time) are centered around every 15th meridian, stretching half a sun hour (7.5 longitudinal degrees) to the east and half a sun hour to the west. Prague for example, is one sun-hour to the east of Greenwich, St. Petersburg is 2 h and Bagdad is three. The opposite side of Earth from Greenwich forms the date line and conveniently consists almost exclusively of Pacific Ocean water.

We will repeatedly refer to three different clocks or time frames here. (i) The *sun clock* shows the local time of the apparent progression of the sun; noon being when the sun is highest and midnight being exactly half way between dusk and dawn. (ii) The *social clock* shows the local time determined by policy in form of devices on walls, on wrists or in phones; it is a social construct referring to the sun time at the meridian that was chosen for that time zone (see below). (iii) The *body clock* determines the organism’s internal time as defined by the circadian clock. Almost all physiological functions from reading certain genes and activating certain proteins to cognitive capabilities and the time when an individual sleeps best are determined by the *body clock* (Borbély et al., 2016). *Body clocks* are predominantly set by light and darkness (Roenneberg et al., 2003a) and can adopt a highly individual relationship to the *sun clock* (e.g., to dawn). The light from the *sun clock* is a stronger stimulus than artificial (e.g., electric) light; we will discuss this more later. We will return to how these clocks synchronize to the solar day below.

If you live directly on an hour-meridian, the *social clocks* at these locations report *sun time*. At all times in any other location, *social clocks* only report the social constructs referring to the sun time at the meridian that was chosen for that time zone. Although time zones were meant to span one sun-hour, it should be noted that time zones are often much wider than 15° longitude. For example, when Galicians in north-western Spain look at their *social clock* in winter and it says noon, it is only 10:30 a.m. by the *sun clock*, since Spain has decided to be part of the Central European Time (CET) zone with its meridian running approximately through Prague. China is even more extreme since all *social clocks* are set according to Beijing’s *sun clock* at the country’s eastern edge despite its western edge being almost five sun-hours away. An understanding of time zones is essential for appreciating the effects of DST because **switching to DST is nothing else but assigning the respective location to one time zone further east.** This switch increases the discrepancy between the *sun clock* and the *social clock* by 1 h. This means that for Galicia during summer, it is only 9:30 am by the *sun clock* when *social clock* claims noon, since they now have to live according to the hour-meridian that runs roughly through St. Petersburg.

## DST and the Body Clock

The association between health and the *body clock* depends on the degree of misalignment between *body clock* and *social clock*. To appreciate this association and its changes over time, one has to understand the mechanisms of entrainment that are at the heart of the *body clock* staying in synch with the 24-h environment.

The “master” *body clock* in the *nucleus suprachiasmaticus* of the hypothalamus receives light via the retina and the optic nerves. The neurons of this master clock actively synchronize (or *entrain*) to the environment’s light–dark signals (*zeitgeber*) and in turn provide entraining signals for the circadian clocks in the rest of the body, i.e., the rest of the nervous system as well as peripheral organs and tissues (Saini et al., 2015). This process involves many components, most of which are proteins controlled by genes. The entraining process shows individual variations in the relationship between the *body clock* and the light–dark cycle (e.g., earlier or later) – the colloquial “larks” and “owls,” or *chronotypes* in general. The number of people in a population that belong to different *chronotypes* shows a statistically close-to-normal distribution, usually with a surplus on the late chronotype (“*owls*”) end of the curve (Fischer et al., 2017). The term chronotype has some times been associated with a relatively stable personality trait or with so-called daily preferences (Horne and Ostberg, 1976), whereas we use it here as the *actual* phase (i.e., time) of entrainment. Phase of entrainment can be assessed in different ways both subjectively and objectively. An example for the former is the Munich ChronoType Questionnaire (MCTQ) that uses subjectively assessed sleep and wake times, specifically the midpoint between sleep onset and offset (Roenneberg et al., 2003b) and an example for the latter are dim-light melatonin onset (DLMO) times (Benloucif et al., 2008).

Historically in humans and other seasonally reproducing animals, the *body clock* responded to the number of hours of daylight that corresponded internally to the number of hours the internal circadian signal melatonin is secreted (Bittman et al., 1985; Wehr et al., 2001; Stothard et al., 2017). The nocturnal duration of melatonin expanded in winter and was compressed in summer. With the invention of artificial light, this zeitgeber has been drastically weakened since we now predominantly spend most of our days in buildings, thereby rarely getting full sunlight, and we switch on artificial light after sunset, thereby only being exposed to darkness (but not necessarily total darkness) when we sleep. One consequence of this weakening zeitgeber strength – on the annual level – is that seasonal rhythms like human reproduction have become weaker and possibly only socially driven (Roenneberg, 2004). Another consequence – on a daily level – is that most individuals’ *body clock* entrains to a later time in relationship to the light–dark cycle, except for the very early larks, who may actually become even earlier under weak zeitgebers. This zeitgeber-strength-dependent change in entrainment can be predicted from circadian formalisms (Roenneberg et al., 2003b) and has been shown for both birds (Dominoni et al., 2013) and humans (Wright et al., 2013). As a consequence, the distribution of chronotypes has become later and much wider than in pre-industrialized conditions (Roenneberg et al., 2007a): while the body clock of extreme larks and owls is somewhere between 2 and 5 h apart in the absence of electrical light (Samson et al., 2017a), they are up to 12 h apart in urbanized regions of the industrialized world (Roenneberg et al., 2007a). When the *social clock* does not follow the large delays of the *body clock*, significant discrepancies between these two clocks arise; this so-called *circadian misalignment* can be assessed for some situations by calculating social jetlag (SJL), which is the difference between sleep-timing on work and work-free days (Wittmann et al., 2006). We discuss the importance of SJL later in this paper.

## Virtual Time Zones

**FIGURE 1:**
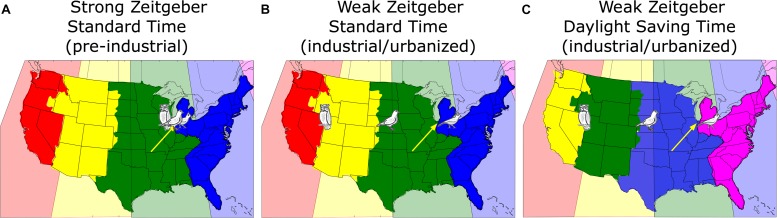
A map of the continental United States showing the actual time zones color-coded in the background, i.e., every 15th longitude to the west of Greenwich ± 7.5° (pink: –4 h; blue: –5 h; green: –6 h; yellow: –7 h; red: –8 h). The political time zones adopted by some areas and states of the United States differ from the physical time zones. The bird drawings reflect the virtual position of three different chronotypes (lark = early; owl = late; dove = intermediate; see text for details) living in Chicago (yellow arrows) under different conditions. **(A)** Pre-industrial without electric light (blue: Eastern; green: Central; yellow: Mountain; red: Pacific); **(B)** in post-industrial times with electric light but under Standard Time; **(C)** as **(B)**, but under Daylight Saving Time, which amounts to a reassignment of the political time zones [note the color change compared to **(A)** and **(B)**].

An individual’s chronotype is usually reported in local time (i.e., by the *social clock*). However, since chronotype is inherently linked with an individual’s light–dark cycle (see above), chronotype is more tightly coupled to the *sun clock* (Roenneberg et al., 2007b). Different chronotypes can therefore be translated to or conceived (in a thought experiment) as living in different longitudinal locations. According to this translation, every chronotype virtually lives in its own chronobiological time zone that – unfortunately in many cases – is different from where the individual actually lives. While all people from Chicago physically live in Chicago [yellow arrows in Figure 1, which was first presented at the Sapporo Symposium 2018 (Roenneberg et al., 2019)], their *body clocks* virtually “live” east or west of Chicago, depending on their chronotype. The discrepancy between the physical time zone and the chronobiological time zone was small when we lived without artificial light as shown in Figure 1A. Corresponding to this virtual time-zone metaphor, the larks “lived” slightly east of the city, the owls slightly west and the intermediate chronotypes (which we will refer to as the “doves”) “lived” near the city center. The average midpoint of sleep (a marker for chronotype) in industrialized/urban areas is around 4 a.m., in pre-industrialized eras it was much closer to midnight (Wright et al., 2013; Samson et al., 2017b; Pilz et al., 2018b).

Under conditions of a weakened zeitgeber and more phase-delaying light exposure in the evening, most people’s *body clocks* have delayed although the earliest larks have advanced, thereby pulling apart the time-zone chronobiology, so that the distribution of *body clocks* that belong to people who live and work in Chicago would look like shown in Figure 1B. We can use the chronotype distribution of the United States population represented in the MCTQ database (*N* = 25,339; the MCTQ database is curated by TR) to translate chronotypes into the equivalent location east-west of Chicago. If we align the median of this distribution (≈ 3:30 a.m.) to the center of Chicago, about 36% of Chicago’s population live both physically and chronobiologically within ±30 min of Chicago’s *sun time*, about 24% would “live” further east, about 18% would live “west,” (matching the longitude of United States state of Nebraska), 12% would “live” even further west between Denver and San Francisco, while another 10% would “live” somewhere in the Pacific between San Francisco and Tokyo. Note that the owners of these *body clocks* have to work in Chicago.

This situation becomes even worse when *social clocks* are switched to DST, which means that the people of Chicago now have to work according to the *sun clock* in Nova Scotia, Canada which is 1 h earlier (pink, illustrated in Figure 1C).

The general delay of the industrialized population’s *body clocks* must be considered when we make decisions about work and school start times. In the pre-industrial era, humans slept mostly between 8 p.m. and 6 a.m. and could easily be at work around 8 a.m. According to the MCTQ database, only 23% United States Americans would – judged by their sleep and wake times on their free-days – wake up before 7 a.m.; the rest would sleep too late relative to needing to be at work at 9 a.m. Not surprisingly, 78% of the working United States population represented in the MCTQ database indicates that they use an alarm clock in winter and 72% in summer; as noted above, *body clocks* are later in winter than in summer, probably because zeitgeber strength is stronger in summer as people spend more time outside (Kantermann et al., 2007; Hadlow et al., 2014, 2018; Hashizaki et al., 2018) and therefore more people would be expected to need alarm clocks in the winter. As one can see in Figure 1A, DST adds an hour to the discrepancy between the *social* and the *body clock* thereby fueling the battle between biological and social time and increasing SJL.

## Myth-Understandings and Confusions Surrounding DST

In summer 2018, the EU asked its citizens to give their opinion about DST in an online poll. 84% of the (predominantly German) participants voted to abolish the twice yearly switch between different clock times; of these a slight majority favored establishing DST all year (European Commission, 2018). Unfortunately, opinions expressed in poll answers and potential decisions based on such opinions may not be based on scientific evidence. In addition, both the lay public and scientists use language in relationship with DST that invites prejudice. For example, in many countries, DST is referred to as “summer time” and Standard Time as “winter time”; the EU poll specifically asked people whether they prefer perennial “summer time” to perennial “winter time.” It is not surprising that more people would chose “summer time” over “winter time.” Other misleading terms are: *we change the time*; *days become longer*; *the sun sets later*; or: *it’s only one hour and we cross many more time zones when we travel* (Table 1). However, with DST, we do not change time, we only change *social clocks*; the *sun clock* with its midday and midnight remains the same and dawn and dusk continue their gradual seasonal photoperiodical/day-length changes. Importantly, days are not becoming additionally longer and the sun does not set additionally later because of DST, we simply come home earlier (in reference to the *sun clock*) because we start work or school earlier (in reference to the *sun clock*). DST changes are not comparable with time changes after transmeridian flight (known as jet lag) because we stay where we are instead of exposing our *body clocks* to the new light–dark cycles of our travel destination.

**TABLE 1:** Short summary of Myth-Understandings surrounding DST, providing short explanations and references.

**Myth-Understandings**	**Explanation**	**References**
Summer Time	The colloquial term is used for DST in many countries, but it is misleading since it implies that DST may be responsible for positive attributes of summer (sunny days, warm temperatures, long days) producing falsely positive connotations. Although DST is mostly during summer months, DST is simply an advance of the social clock (we agree to do everything 1 h earlier) and does not “make it summer”	See text for details
Winter Time	This term used for Standard Time is a consequence of calling DST “Summer Time.” It is just as misleading, because Standard Time refers to the social time defined by the time zone and has nothing to do with winter: it does not “make it winter” nor cause short days, cold temperatures or snow	See text for details
Time Change	This term is misleading because DST does not change time but only the local clock, which is used as reference for local (social) time	See text for details
Under DST, “days are longer” or “the sun sets later”	DST does not change day length or the time of sunset; day length changes with *season* in most parts of the world. During DST, people go to work an hour earlier (relative to sunrise) and come home an hour earlier (relative to sunset)	See text for details
DST is like traveling to the next time zone	It is correct that people can readily adapt to traveling one time zone west or east, but they adapt because their circadian clocks are exposed to the new natural light–dark cycle. DST, however, does NOT change the natural light–dark cycle. DST changes are therefore NOT comparable to traveling to different time zones	See text for details
It is only 1 h	It is true that DST clock-changes are usually 1 h, but the relationship between sunrise and when we start work can change by many weeks. Also, a mismatch of 1 h/day is enough for adverse effects, especially if it lasts chronically for 7 months	See text for details
Negative DST effects in spring are compensated for in autumn	Although the two opposite effects are true epidemiologically, the autumn “relief” cannot rescue the spring victim on an individual level. The spring victims can be only rescued by abolishing the clock advance (DST) Also, the autumn relief indicates the existence of a stressor prior to the clock change, even if that stressor is “merely” chronic sleep deficiency	Manfredini et al., 2018 See text for details
Sun time plays only a minor role for the body clock since it is mainly set by artificial light in modern humans. Environmental temperatures play also a role	The human circadian clock can be set by both sunlight and artificial light, but sunlight is usually up to 1,000-fold more intense and has been shown to affect the clock’s synchronization even in mostly indoor-living people. There is no evidence for clock synchronization by environmental temperature in humans (although sleep times may be affected)	Lucas et al., 2014; Roenneberg et al., 2007b; Buhr et al., 2010; Hadlow et al., 2018
The effects of DST can be neglected compared to those elicited by the use of smart phones	Use of smart phones in the evening can delay the body clock. However, this effect does not compete with the light effects of DST, on the contrary, the two act additively in the same direction, thereby worsening social jetlag (SJL) Evening light delays the clock and morning light advances the clock. Thus, DST decreases circadian clock advances and increases circadian clock delays	Cajochen et al., 2011; Chang et al., 2015; Chinoy et al., 2018; Roenneberg et al., 2003a
Perennial DST does not significantly increase SJL	The reverse is true: the number of people NOT suffering from SJL doubles when switching away from perennial DST to Standard Time (e.g., time zone time) and the number of people suffering from higher levels of SJL are significantly and greatly reduced There have been multiple attempts to implement perennial DST over the past 100 years (e.g., in Russia, the United Kingdom and the United States). In each of these cases, the “experiment” was abandoned after few years	Borisenkov et al., 2017; Ripley, 1974; Uk Time Zone History, 2019; Gray and Jenkins, 2018

People often belittle the effects of DST by stressing that “it’s *only one hour.*” Note that this 1 h can actually translate into throwing our body clock’s relationship to social clock back weeks in the seasonal changes between sunrise and work start time (Kantermann et al., 2007): in mid-winter (before we switch to DST), most people at higher latitudes (e.g., further from the equator) get up on workdays before sunrise; weeks later in spring (during Standard Time, still before the DST switch), they start getting up with the sun and then in the following weeks, the sun gets up before them. However, when the switch to DST occurs, they are getting up before the sun again, throwing the relationship between sunrise and get-up back by three weeks or more (depending on latitude) in their seasonal trajectory. The question is, however, what happens if this mismatch of 1 h is maintained throughout the DST period (see below)?

In September 2018, two sleep researchers from the University of Salzburg claimed in an interview that there is no hard scientific evidence against perennial DST and that the risks would be negligible (Press Release University of Salzburg., 2018). This press release contained several statements that echo wide-spread incorrect beliefs and is therefore an excellent substrate for clarifying fallacies (Table 1).

Their first fallacy refers to reported acute effects of DST: “*Sleep problems, performance deficits, and even increased risks for cardiac infarction are reported, effects that are however equalized in autumn.*” This refers to a paper published in the New England Journal of Medicine (Janszky and Ljung, 2008) showing a relative increase of myocardial infarction after the spring DST change and a relative decrease after its release in autumn, and the many studies reporting increased myocardial risk just after the switch to DST in the spring have recently been reviewed (Manfredini et al., 2018). The above statement by the Salzburg researchers is misleading in two ways. First, the spring and the autumn effect do not balance each other out on the *individual* level and the higher risk in spring is avoidable by abolishing DST. Second, the paper by Janszky and Ljung show a decrease in risk on the days immediately following the autumn release from DST. If physiology had fully adapted to DST and if the decreased cardiac risk was not only from the extra hour of sleep the night DST ends, this decrease should not occur. Thus, the results of this paper suggest that the risk for myocardial infarction was elevated throughout DST.

The second fallacy concerns entrainment: “*The endogenous clock is predominantly but not exclusively set by sunlight, artificial light and environmental temperature play also a role*.” It is true both that light is the major zeitgeber for the circadian clock, and that there is no reason to separate sunlight from other light sources: as long one can define the light’s intensity and spectral composition one can make predictions about its strength to entrain (Lucas et al., 2014). In contrast, entrainment by external temperature changes is most likely not relevant in mammals (Buhr et al., 2010). A paper investigating sleep in pre-industrial societies (Yetish et al., 2015) claims that the tribes they studied are awoken by cold morning temperatures. But cold morning temperatures *per se* do not *entrain* the human clock and are thus more comparable to an alarm clock than to a zeitgeber.

The press release adds that “… *many people extensively use smart phones or laptops shortly before they go to bed. The strong blue components … are the true robbers of sleep. … the potential effects of summer time can be neglected in comparison*.” Indeed, several studies have shown that the usage of artificial light in the evening, and specifically that of electronic screens, does “*rob*” sleep and delays the circadian clock (Cajochen et al., 2011; Chang et al., 2015; Chinoy et al., 2018). The second half of the statement, however, is missing the point: the combination of nighttime light exposure and DST is far worse than nighttime night exposure alone. The nighttime light exposures delay the *body clock* in relation to the *sun clock* (Roenneberg et al., 2003a), which translates to living further west is a time zone, while DST advances the *social clock* in relation to the *sun clock*, which translates into moving the time zone further east. Thus, in DST, the two effects additively (i.e., both delaying the *body clock* and advancing the *social clock* in relation to the *sun clock*) increase SJL since both effects increase the difference between the mid-sleep on work-free days (closer to the individual circadian mid-point of sleep) and mid-sleep on workdays.

The last fallacy concerns the interpretation of data in a paper published by Russian researchers analyzing the three different *social clock* constructs in Russia’s recent history (Borisenkov et al., 2017): the traditional DST pattern of usage in only some months of the year, the extension of DST to the entire year (which was abandoned after 4 years) and the subsequent permanent Standard Time (which the government finally adopted after going through these different nation-wide “experiments”). The researchers showed that SJL gradually increased from perennial Standard Time to traditional DST/Standard Time switching to perennial DST. In reference to the 2017 Borisenkov paper, the Salzburg sleep researchers said: “*At first glance, the study seems to support … that permanent summer time fosters social jetlag. If you look at the results carefully, the reported effects are very small.*” When analyzing the results of a population-based study, it is always beneficial to look at the actual distributions of results rather than changes only in average results of the population. When we do this, we see that permanent DST would increase the average SJL by more than half an hour which may be statistically small but is biologically large. The distributions published by Borisenkov and colleagues show that the transition from perennial DST to perennial Standard Time led to doubling of people who do not suffer from SJL, those who suffer from only 1 h SJL increased by about 30% and those who suffer from higher SJL are reduced by 25%. Therefore, Standard Time reduced SJL.

Russia was not the only country to try and then abandon permanent DST. The United States has tried it twice (one after WWII and one in the 1970s) and the United Kingdom tried it in the 1970s. Expected energy savings were not observed in the United States or elsewhere, and in both the United States and the United Kingdom, permanent DST was highly unpopular (Ripley, 1974; Uk Time Zone History, 2019). The US Congress even ended the 1970s DST plan early because of its unpopularity. A revealing review of the political considerations within the United States of the DST laws is available in Gray and Jenkins (2018); farmers, parents of children who waited for school buses in the dark, and people in higher latitudes were especially vocal against permanent DST. In the United States, minimal or no energy savings were observed (as noted above), and there were some increases in traffic accidents in the morning. Two interesting additional political facts from this Gray and Jenkins reference include: (i) one group lobbying for more months of DST were candy manufacturers and “concerned” parents who wanted Halloween Trick-or-Treating to happen during sunlight hours; and (ii) members of Congress living on the western portion of each time zone were less likely to vote for DST, as would be expected from the biology we have been describing.

## Time Zones and Health

There are good sides to DST, such as coming home “earlier” (by the *sun clock* but not by the s*ocial clock*) from school or work and therefore having more hours of daylight during the free time after work. These positive effects may go beyond subjective feelings. A study has shown for example that activity increases with longer evening daylight (Goodman et al., 2014) – albeit with small biological effect sizes (≈6% difference in the daily activity between the Standard Time of the year and DST, adjusted for photoperiod). Interestingly these results of the above study were culture-specific: a significant increase was mainly observed in Europe and to some extent in Australia, while no significant effects or even slightly negative effects were seen in the United States and Brazil.

It is important to note that DST transitions can elicit short and/or long-term effects, which we will refer to as *acute* and *chronic* effects, respectively. The first days after the DST change in spring show *acute* effects: sleep is shortened (Barnes and Wagner, 2009), adolescents are sleepier during the day (Schneider and Randler, 2009), general accidents and visits to the emergency room increase (Ferrazzi et al., 2018), so do myocardial infarctions (Janszky and Ljung, 2008; Manfredini et al., 2018), ischemic stroke (Sipilä et al., 2016), the risk of *in vitro* fertilized mothers losing their babies (Liu et al., 2017), and suffering from negative mood changes (Monk and Folkard, 1976; Monk and Aplin, 1980). In these last two papers, the authors suggest that the effects of DST are similar to those of shift-work, which has known multiple adverse effects on health and safety due to the mismatch between the *body clock* and the *social/work clock* (Scott, 2000; Knutsson, 2003). On the Monday after the DST transition, the known stock market weekend effect (i.e., a predictable negative influence on stock-trading each Monday morning), is augmented by 200–500% in several international markets, implying a $31 billion one-day loss in the United States markets alone (Kamstra et al., 2000). Reports have been contradictory on the *acute* effects of DST on traffic accidents (Carey and Sarma, 2017). Some find an increase after transitions to and from DST (Hicks et al., 1983), others find an increase in spring (to DST) and a decrease in fall (from DST) (Coren, 1996a,b), some find no change (Huang and Levinson, 2010; Lahti et al., 2010) or even the reverse effect (Ferguson et al., 1995) though this last paper used as their reference the light patterns for one city in the center of the United States rather than those where the accident occurred. Similarly contradictory are reports on hospital admissions. While a Finish study finds no effects (Lahti et al., 2008), an Italian study (Ferrazzi et al., 2018) finds a significant increase in spring and a decrease in autumn. One possible explanation for the different results is that the effects may be latitude-dependent and/or depend on the exact time point of the DST change in relationship to photoperiod, since *acute* DST effects will be different whether the switch is before, at, or, after the spring equinox (note, the date of DST switch changes from year to year and can differ by weeks between different countries).

There are very few reports on the *chronic* effects of DST. These are difficult to study as comparison between periods with DST and Standard Time are usually inherently confounded by seasonal effects. The *chronic* effects may be small on an individual level, but they accumulate over time in individuals and both across time and space in populations resulting in big effects, the costs of which can be assessed similarly to those of insufficient sleep (Hafner et al., 2017). From a chronobiological perspective, *chronic* effects are very likely because, throughout the months of DST, *body* and *social clocks* are likely set to different time zones in most people, as we explained above. Due to the fact that mostly only the transitions to and from DST in spring and autumn have been studied rather than the steady state effects in summer and winter it is still unclear whether and how much people actually adjust to DST through artificial light exposure. When daily sleep timing as well as activity profiles are recorded for several weeks before and after the transitions, one can see that sleep times adapt relative quickly to the new social time regime – especially on workdays (Kantermann et al., 2007) – while activity profiles on work-free days seem to be relatively insensitive to the DST change hinting at no or very slow adjustment of daily activity rhythms to DST (Roenneberg et al., 2013). An *increased* mismatch between *body clock* and *social clock* time during DST is supported by the only two published steady-state studies that we know of. In the first study performed in Australia, cortisol rhythms were found to be advanced by only 2 min during DST (not the 1 h corresponding to full adjustment) when comparing 3,000 summer samples taken during a 3-year-DST-test phase versus 9,000 summer samples during Australian non-DST summers (Hadlow et al., 2014). This finding suggests no change in *body clocks* despite the change in *social clock* during DST. This lack of change in the *body clocks’* timing during DST compared to during Standard Time demonstrates that our *body clocks* do not heed *social clocks* because *body clocks* are based on *sun clocks* and not political laws; political laws cannot determine health – they can only influence it for the better or worse. In the second study, the analysis of the three different states of DST in Russia (i.e., traditional switching, perennial DST and perennial Standard Time) found an increase in SJL during perennial DST (see above) (Borisenkov et al., 2017). The same study also found a small decrease in winter depression symptoms during perennial Standard Time (Borisenkov et al., 2017). As mentioned above, any study showing long-term positive effects with the cessation of DST in autumn suggests that *chronic* negative effects have likely been acting throughout the months of DST. Even if the positive effects are due to sleep extension on the one night of the DST-to-Standard Time transition, they would indicate a prior sleep debt during DST (Klerman and Dijk, 2005).

In addition to these few studies that address chronic DST effects directly, there are four indirect ways of estimating *chronic* DST effects: (i) relative position in a time zone (i.e., distance from the eastern border of the time zone as indicated in Figure 1; (ii) SJL; (iii) being a late chronotype; and (iv) sleep-loss.

(i) *Relative position in time zones.* Several studies have investigated the prevalence of different cancer types as well as general and cancer-specific mortality as a function of distance from the eastern border of the time zone: (Borisenkov, 2011; Gu et al., 2017; VoPham et al., 2018). All three studies conclude that risks increase and longevity decreases from the eastern to the western border of time zones. The most recent example of studies that examine east-west gradients in time zones (Giuntella and Mazzonna, 2019) finds that “an extra hour of natural light in the evening reduces sleep duration by an average of 19 min” with significant effects on health (e.g., obesity, diabetes, cardiovascular diseases, and breast cancer) and on economic performance (per capita income).

These results suggest that the discrepancy between the *social clock* and the *sun clock* even within a time zone can have a significant effect on health; on the western edge of a time zone, *social clock* time is later than *sun clock* time (as is the case during DST) and at the eastern edge it is earlier. Similar findings are reported for the incidence of winter depression, which also gradually increases within time zones, i.e., the later the sun rises in reference to the social clock (White et al., 2006). This is in contrast to Olders (2003), who finds that later sunrise times, i.e., more western positions in the time zone, are associated with a lower depression prevalence in urban populations. The author argues that sleeping late increases REM sleep, and thus may increase depression risk and suggests that later sunrise times mean earlier rising times in relation to sunrise and therefore proposes to switch to perennial DST. If one would simply extrapolate the results shown in their Figure 1, an increase in average sunrise time of about 1 h would decrease depression prevalence by 50%. This would be a huge effect and has not been documented and would also predict that depression prevalence in winter should be >50% lower than in summer (which is the opposite of what is reported). It is also contrary to the well-documented, beneficial effects of early morning light in mood disorders (Wirz-Justice and Benedetti, 2019). Also, in their Figure 1, which is based on 1-year averages, demonstrates only an association that does not allow causal inference. Furthermore, there are many confounding variables that can affect the prevalence of depression (such as age, sex, socioeconomic status, etc.) that were not adjusted for in the analysis, which may have led to this unexpected finding of an association between lower depression prevalence and later sunrise times. Later sunset times are also associated with fewer hours of sleep, poorer academic performance, and lower wages among adults in a study of people in India, Indonesia, and China (Jagnani, 2018).

**FIGURE 2:**
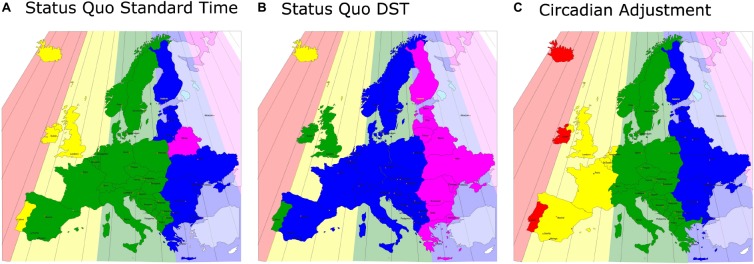
A map of Europe equivalent to Figure 1: the actual, sun-based time zones are drawn as color-coded backgrounds and the social time zones are shown in the same (stronger) colors in front. Even under Standard Time, the western areas of the social time zones are far away from the respective eastern borders of the sun-based time zones **(A)**, this discrepancy increases by 1 h under DST **(B)** (note that Iceland is on perennial DST). **(C)** A solution to the problem: the political borders of Europe are actually ideal for the correct, chronobiological separations into time zones, so that in no area of Europe the social clock has to be discrepant from the sun clock by more than 30 min.

**FIGURE 3:**
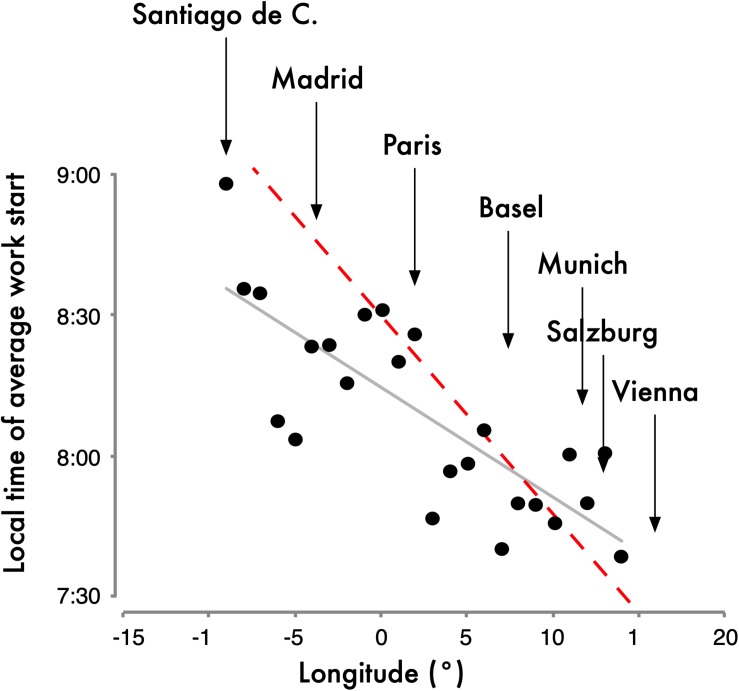
Work-start times averaged for longitudinal bins of the Central European Time (CET) Zone. Data were taken from the MCTQ database. For the analysis, participants had to live in the CET zone and their postal code and city name had to match as they were used to derive latitude and longitude. The dashed red line represents the slope parallel to the progression of sunrise (4 min/longitude). The slope of the actual east-west delay (gray line) in work start times is 2.3 min/longitude (n = 24; r^2^ = 0.63; p < 0.001). This delay in work-start times despite identical local times is noteworthy since the slope of sleep timing on work-free days is 3.8 min/longitude for rural regions, 2.6 min/longitude for towns with populations between 300,000 and 500,000 and 1.5 min/longitude for the major European cities, perhaps due to the differences in lighting patterns and zeitgeber strength in those environments (Roenneberg et al., 2007b). The geographical location of some major European cities is indicated above the graph. Note that while all cities listed here are currently in the CET zone, some of those cities’ longitude is outside the non-political longitude lines for CET and therefore the graph has cities from –15° to +15° instead of –7.5° to +7.5° (see color-coded time zones in Figure 2). Data originally published in Roenneberg et al. (2019).

**FIGURE 4:**
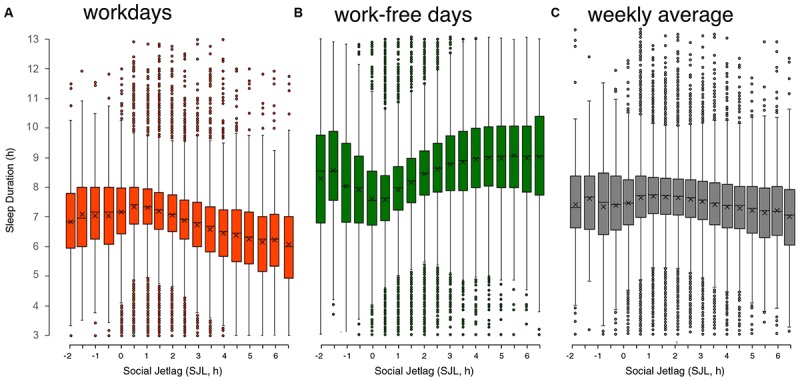
Relationship of sleep duration on workdays **(A)**, on work-free days **(B)**, and weekly average sleep duration **(C)** with social jetlag (SJL). Data are taken from the MCTQ database (*n* ≈ 300,000). Participants with a complete set of questions necessary to compute SJL and with the respective MCTQ variables being within three standard deviations of the mean (μ ± 3σ) were used for the analysis (workdays: *n* = 193,927; work-free days: *n* = 198,096; weekly average: *n* = 193,927). On a weekly average, people who suffer from 3 h of SLJ or more sleep approximately 1 h less per night, adding up to a sleep-loss of 5 h/week. Boxplots are Tukey boxplots with whiskers encompassing all data within 1.5 times the interquartile range; data outside these ranges are depicted as points. ANOVA shows that for free days and workdays significant differences are found on average 3–4 half-hour bins apart while for the weekly average significance is only reached at differences of 7–8 half-hour bins (data originally published in Roenneberg et al., 2019).

The health effects that depend on the east-west position within a time zone described above have not yet been investigated in the CET zone. Of interest, results from the MCTQ database from the CET zone indicate that the expected negative effects may be countered by compensatory behavior. The further west people live within the CET zone, the later they organize their lives in reference to *social* time. Anecdotal evidence suggests that Germans dine later than Hungarians, the French eat later than Germans and Spaniards go to dinner later than the French. Note that France and Spain strictly speaking should not be in the CET since their longitude is further west (see Figure 2); if their time zones were matched to their longitudes, they would not be eating “late” by *social clock* time [because 9 p.m. CET is at the same time as 8 p.m. Greenwich Mean Time (GMT)]. More quantifiable questionnaire results from the MCTQ support this anecdotal evidence and also show that work-start times become progressively later in Europe from its eastern to its western border (Figure 3; Roenneberg et al., 2019). These behavioral changes mean that people eat and work closer to their *body clock* time (rather than the *social clock* time). If the negative east-west health effects shown for Russia, China and the United States turn out to be smaller in Central Europe, it could be due to this compensatory behavior and the east-west slope would be greater if the SJL was not reduced by compensatory behavior.

(ii) *Social jetlag (SJL)*. That human *body clocks* entrain to light–dark cycles as circadian clocks do in all other animals and plants is still true for industrialized societies (Roenneberg et al., 2007b). DST increases the discrepancy between the *sun clock* and the *social clock* and will therefore also increase the discrepancy between the *body clock* and the *social clock*, thereby also increasing SJL (see above). SJL is associated with adverse health effects: these include increased likelihood to be a smoker as well as higher caffeine and alcohol consumption (Wittmann et al., 2006); higher incidence of depression (Levandovski et al., 2011) and other mood pathologies such as anxiety disorders and personality disorders (Wittmann et al., 2010; Foster et al., 2013); increased risk of metabolic disorders (Rutters et al., 2014; Parsons et al., 2015), such as obesity (Roenneberg et al., 2012), metabolic syndrome and type II diabetes (Koopman et al., 2017) or increased insulin requirements in adolescent diabetes-type-I patients (Schnurbein et al., 2018); higher rates of cardiovascular problems (Wong et al., 2015) and cognitive performance and academic achievements (Haraszti et al., 2014; Díaz-Morales and Escribano, 2015).
(iii) *Late chronotype.* There is a strong correlation between chronotype and SJL (Wittmann et al., 2006), which is not surprising since two factors come together in generating the modern condition of having a late *body clock* and having to get up earlier than the *body clock* would suggest. Being a late chronotype is associated with reduced health (Partonen, 2015), but it is most likely that most of the associations between chronotype and health act via SJL rather than via chronotype itself (Levandovski et al., 2011; Pilz et al., 2018a). Since DST would delay chronotypes (see above), any association between late chronotypes and reduced health would be an indicator of *chronic* DST effects.
(iv) *Sleep-loss. SJL* and circadian disruption are strongly correlated with a reduction in sleep duration (Foster et al., 2013). Accordingly, the MCTQ database shows a systematic association between SJL and sleep duration (Figure 4). The SJL-dependent sleep loss during the workweek (Figure 4A) is almost compensated for on work-free days (Figure 4B), so that SJL is characterized by a constant oscillation between under- and over-sleeping. Notably people suffering from less than 30 min of SJL get the longest sleep on workdays and sleep the least on their free days compared to those of other SJL categories. Access to electrical light itself is associated with a decrease in sleep duration (de la Iglesia et al., 2015; Pilz et al., 2018b), which can be explained by a concurrent delay of chronotype (Wright et al., 2013; Pilz et al., 2018b). Of the almost 200,000 people represented in Figure 4, only 12% do not suffer from SJL and another 19% suffer from only half an hour of SJL. These two categories are the only ones that get on average more than 7 h of sleep on nights before workdays, all other chronotypes gets less than 7 h of sleep as SJL increases. Notably, sleep deficiency is associated with the same health risks as DST, SJL and being a late chronotype, e.g., with metabolic pathologies (Borel et al., 2013; Reutrakul et al., 2013, 2015), suggesting that the effects have common mechanisms, for which sleep debt could be a good candidate.

## Potential Solutions

In summary, the scientific literature strongly argues against the switching between DST and Standard Time and even more so against adopting DST permanently. The latter would exaggerate all the effects described above beyond the simple extension of DST from approximately 8 months/year to 12 months/year (depending on country) since *body clocks* are generally even later during winter than during the long photoperiods of summer (with DST) (Kantermann et al., 2007; Hadlow et al., 2014, 2018; Hashizaki et al., 2018). Perennial DST increases SJL prevalence even more, as described above.

A solution to the problem is shown in Figure 2C, which contains a combination of obliterating DST (in favor of permanent Standard Time) and reassigning countries and regions to their actual *sun-clock* based time zones. Under such adjustment, *social (local) clock* time will match *sun clock* time and therefore *body clock* time most closely. Critics of such a solution might argue that this would scatter European social times, but there is no evidence that this would be detrimental. First, we already have three different time zones within Europe (WET/GMT, CET, and EET), and secondly, the United States has four different time zones and several United States states even have multiple time zones with no detriment in commerce, travel, or communications.

If DST should be abandoned, as we suggest as scientists, there are still many people who “like their long evenings.” But there is a solution to this problem: DST is simply a work-time arrangement, nothing more than a decision to go to school/work an hour earlier. As such, it is not a decision that should be made by the world, by unions of countries (e.g., the EU), or by individual countries, neither at the federal nor the state level. Work-time arrangements are decisions that a workforce could decide at the company level. Therefore, anyone who wants to spend more time at home in daylight after work should convince his/her company and co-workers to advance their start time during certain months of the year or even better: introduce flexibility for individual workers where possible to accommodate differences in personal biological and social requirements.

## Summary

Discrepancies and misalignments between *social (local) clock* time, *sun clock* time, and *body clock* time can be caused by political decisions: DST is one example. There are multiple health and safety consequences of these misalignments. Our goal is that this article’s facts and reasoning will be used to make clock choices that improve human lives.

## Author Contributions

TR wrote the first draft of the manuscript, performed analyses of the MCTQ database, and generated the first version of the figures. EK and EW contributed to the conceptual content of the manuscript and co-wrote the manuscript following the first draft.

## Conflict of Interest Statement

The authors declare that the research was conducted in the absence of any commercial or financial relationships that could be construed as a potential conflict of interest.
