# Corrigendum: Daylight Saving Time and Artificial Time Zones – A Battle Between Biological and Social Times

**DOI:** 10.3389/fphys.2019.01177

**Published:** 2019-09-12

**Authors:** Till Roenneberg, Eva C. Winnebeck, Elizabeth B. Klerman

**Affiliations:** ^1^Institute of Medical Psychology, Ludwig Maximilian University of Munich, Munich, Germany; ^2^Division of Sleep and Circadian Disorders, Brigham and Women's Hospital, Harvard Medical School, Boston, MA, United States

**Keywords:** circadian, social jetlag, circadian misalignment, time zones, entrainment (light)

In the original article, there was a mistake in Figure 2B as published. In the map, the area of Germany was duplicated and appeared south-west of Norway. The corrected Figure 2 appears below.

**Figure 2:**
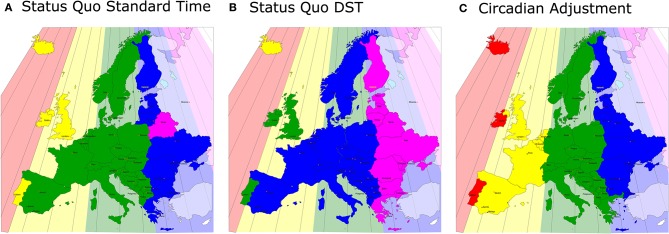
A map of Europe equivalent to Figure 1: the actual, sun-based time zones are drawn as color-coded backgrounds and the social time zones are shown in the same (stronger) colors in front. Even under Standard Time, the western areas of the social time zones are far away from the respective eastern borders of the sun-based time zones **(A)**, this discrepancy increases by 1 h under DST **(B)** (note that Iceland is on perennial DST). **(C)** A solution to the problem: the political borders of Europe are actually ideal for the correct, chronobiological separations into time zones, so that in no area of Europe the social clock has to be discrepant from the sun clock by more than 30 min.

The authors apologize for this error and state that this does not change the scientific conclusions of the article in any way. The original article has been updated.

